# Sacroiliac Stretching Improves Glenohumeral Internal Rotation Deficit of the Opposite Shoulder in Baseball Players in a Randomized Control Trial

**DOI:** 10.5435/JAAOSGlobal-D-18-00060

**Published:** 2018-10-08

**Authors:** Victor Romano, Joseph Romano, Gregory E. Gilbert

**Affiliations:** From the Romano Orthopaedics Center, Oak Park, IL (Dr. V. Romano), the Loyola University Medical Center, Maywood, IL (Dr. J. Romano), the Learning Sciences, Adtalem Global Education, Downers Grove, IL (Dr. Gilbert), and the Center for Teaching and Learning, Ross University School of Medicine, Roseau, Commonwealth of Dominica, West Indies (Dr. Gilbert).

## Abstract

**Introduction:**

Glenohumeral internal rotation deficit (GIRD) is a well-documented finding in throwing athletes.

**Purpose:**

To investigate whether stretching the contralateral sacroiliac (SI) joint can improve GIRD in baseball players.

**Method:**

After internal shoulder rotation was measured in 23 minor league baseball players, the players randomly were assigned to either a control (ie, sleeper stretch of the dominant shoulder) or experimental (ie, SI joint stretch contralateral to the dominant shoulder) group. Afterward, internal rotation (IR) of their dominant shoulders was remeasured.

**Results:**

The mean initial end-range IR was 68.6° (SD = 7.9°) in the sleeper stretch group (n = 8) and 64.5° (SD = 5.1°) in the SI joint stretch group (n = 15). After stretching, the sleeper stretch group's mean end-range IR was 72.1° (SD = 7.2°), a 3.5° improvement (*P* = 0.1058), whereas the contralateral SI joint stretch group's mean end-range IR was 71.9° (SD = 6.6°), a 7.4° improvement (*P* = 0.0041).

**Conclusions:**

Stretching the contralateral SI joint improved GIRD more than the sleeper's stretch.

The loss of shoulder internal rotation (IR) in the overhead thrower has been referred to as glenohumeral internal rotational deficit (GIRD) and was originally described by Burkhart et al.^1^ Although its etiology is unclear, GIRD is a common physical impairment found in overhead athletes.^2,3,4,5^ Immediately after pitching, pitchers often demonstrate a loss of shoulder IR lasting over 24 hours.^6,7^ Pitchers with GIRD have up to a four times higher incidence of shoulder and/or elbow injuries and miss more games during the baseball season compared with pitchers with normal range of motion.^8-10^ Furthermore, stretching the shoulder and restoring full range of motion have been shown to lower the rate of injuries in these athletes.^10,11^

The optimal method for increasing shoulder range of motion (ROM), improving performance, and preventing injury is unknown. Many stretching programs have been studied with varied results.^12,13,14,15,16,17,18,19,20^ The most common of which are the sleeper and cross-body adductor stretches. Laudner et al^12^ demonstrated that the sleeper stretches produced a statistically significant improvement of rotation; however, it is unclear whether their 3.1° improvement is clinically significant. McClure et al^14^ found that both sleeper and cross-body stretches improved IR and that the cross-body stretch was slightly superior; however, their study was underpowered to show a statistical difference between the two stretches. Additional studies demonstrate improved shoulder range of motion with both stretching protocols but have failed to establish a statistically significant superiority of one regiment over the other.^13,16,18,20^

During physical examination and observation of pitching mechanics in overhead pitchers with shoulder pain, the principal investigator has found a correlation between the loss of IR of the shoulder and tilting of the contralateral pelvis. In addition, it has been further observed that resolving the pelvic tilt by stretching the sacroiliac (SI) joint improves IR of the contralateral shoulder. The purpose of this study was to investigate whether stretching the contralateral SI joint improves GIRD in overhead athletes. In addition, we aimed at comparing our SI joint stretching regiment with a classically described sleeper stretch routine.

## Methods

This study obtained institutional review board approval from our institution. It was designed as a double-blinded pilot study of minor league baseball players. Inclusion criteria were being men, aged 23 to 25 years, and a member of a Midwestern minor league baseball team. Data were collected on all 23 subjects 2 days before their season-opening game. All participants were healthy, without shoulder pain, and with no history of shoulder injury. The study was conducted in the team's training room, one at a time, after we obtained informed consent without explaining our hypothesis. No players were paid for participation.

**Figure 1:**
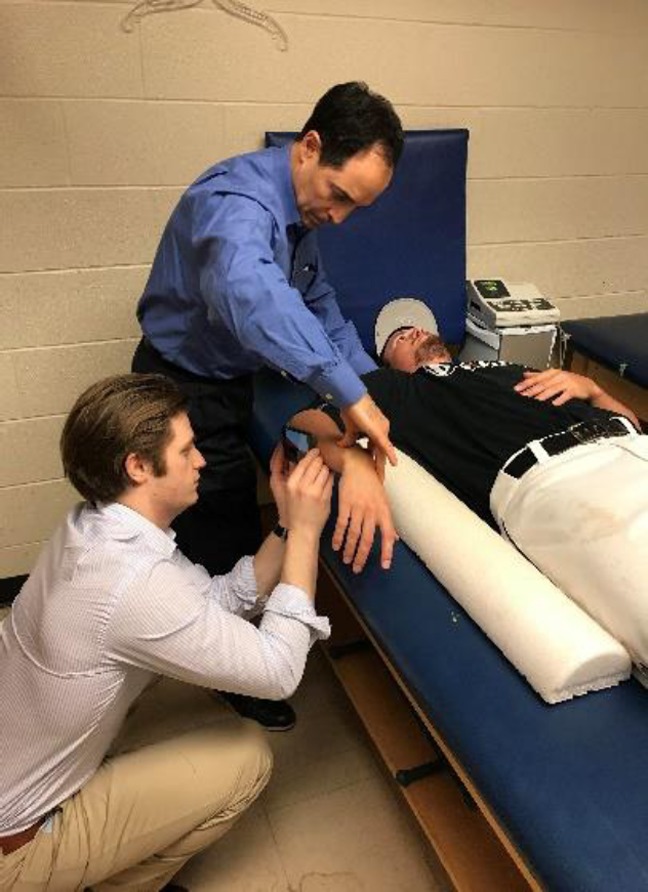
Photograph showing measurement of internal rotation with the compass app on iPhone.

With the player supine on the examination table with his arm abducted 90°, a wedge was placed under the shoulder to position the arm in the scapular plane (Figure 1). Working together, two investigators measured the end-range IR of both shoulders. One investigator stabilized the scapula by holding the coracoid process with the thumb and the spine of the scapula with his fingers while maximally internally rotated the shoulder.^21^ The other investigator measured the angle of the forearm from the horizon using the horizontal leveler on the Apple iPhone Compass app. The horizontal axis of the iPhone was aligned with the olecranon and shaft of the ulna toward the ulnar styloid. The angle between the forearm and the horizon was measured. Internal ROM was determined by calculating the complementary angle of the measurement. Each measurement was performed twice, and the lowest measurement was recorded. If the measurements were off by more than 3°, a third measurement was made and recorded.

Next, the player went to a second examination room where an independent medical assistant randomly assigned the player to either a control or an experimental group via an online random number generator. Each group was supervised by a certified athletic trainer because the players did their own stretches. The athletic trainers did not know the results of the players' measurements. In the control group, the players stretched their dominant shoulder via the sleeper's stretch. Lying on their dominant side with their backs stabilized against the wall and their dominant arms abducted 90°, they forcefully brought their forearms toward the table using their opposite arm. Each stretch was held for 30 seconds for a total of three stretches.

In the experimental group, the players stretched the opposite SI joints of their dominant shoulders. Lying supine, each player grabbed the knee opposite his dominant shoulder and forcefully brought it up toward his dominant shoulder. He then brought his knee across his dominant chest while fully abducting the nondominant arm and shoulder in the opposite direction. Again, each stretch was held for 30 seconds for a total of three stretches.

Finally, each player returned to the first examination room. The initial two investigators, unaware of to which group the player was assigned, remeasured the end-range IR of his dominant shoulder.

Descriptive statistics were calculated for the players participating in the study. Because of the small sample size, nonparametric statistics were used for inferential analyses (Wilcoxon signed-rank test).

In a separate analysis, interrater and intrarater reliability among the examiners were determined for assessing the range of motion using the compass app. Three examiners measured the IR of both shoulders in four healthy volunteers with no history of shoulder injuries. With the subject supine on the examination table with his or her arm abducted 90°, a wedge was placed under the shoulder to position the arm in the scapular plane. The subject relaxed and the arm was allowed to maximally internally rotate via gravity. No force was used. Working together in groups of two, one investigator stabilized the scapula, whereas the other investigator measured the angle of the forearm from the horizon using the horizontal leveler on the Apple iPhone Compass app. Again, the horizontal axis of the iPhone was aligned with the olecranon and shaft of the ulna toward the ulnar styloid. However, the numbers on the iPhone screen were covered by electrical tape and could not be seen by the examiners. To record the data, a screenshot of the compass app was taken after each measurement and was not accessed until after the study was completed. Each examiner made five separate measurements of both shoulders in each subject. After each measurement, the subject rested, and his or her shoulder was repositioned.

## Results

We randomized all 23 minor league baseball players to sleeper stretch (n = 8) and contralateral SI joint stretch groups (n = 15) (see Supplemental Table 1, http://links.lww.com/JG9/A25). No players were excluded from this study. Group demographics (ie, age, position, and arm dominance) were similar. The mean initial end-range IR was 68.6° (SD = 7.9°) in the sleeper stretch group, with a mean contracture of 5.3° (SD = 10.3°), compared with the nondominant shoulder. In the SI joint stretch group, the mean initial end-range IR was 64.5° (SD = 5.1°), with a mean contracture of 10.3° (SD = 9.5°).

**Figure 2:**
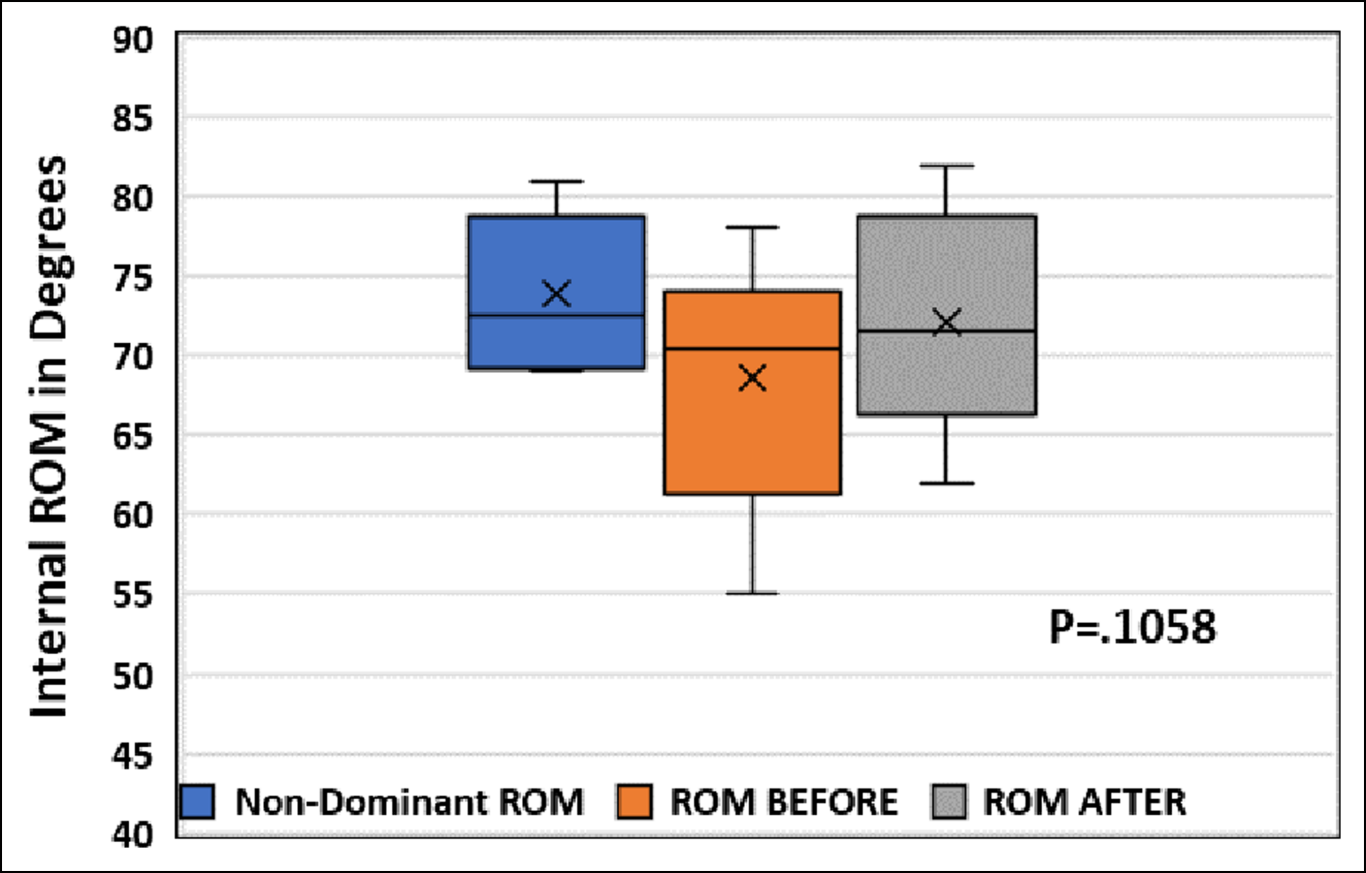
Box and whisker plot showing shoulder internal rotation of eight minor league baseball players on the nondominant arm and dominant arm before and after sleeper stretch.

**Figure 3:**
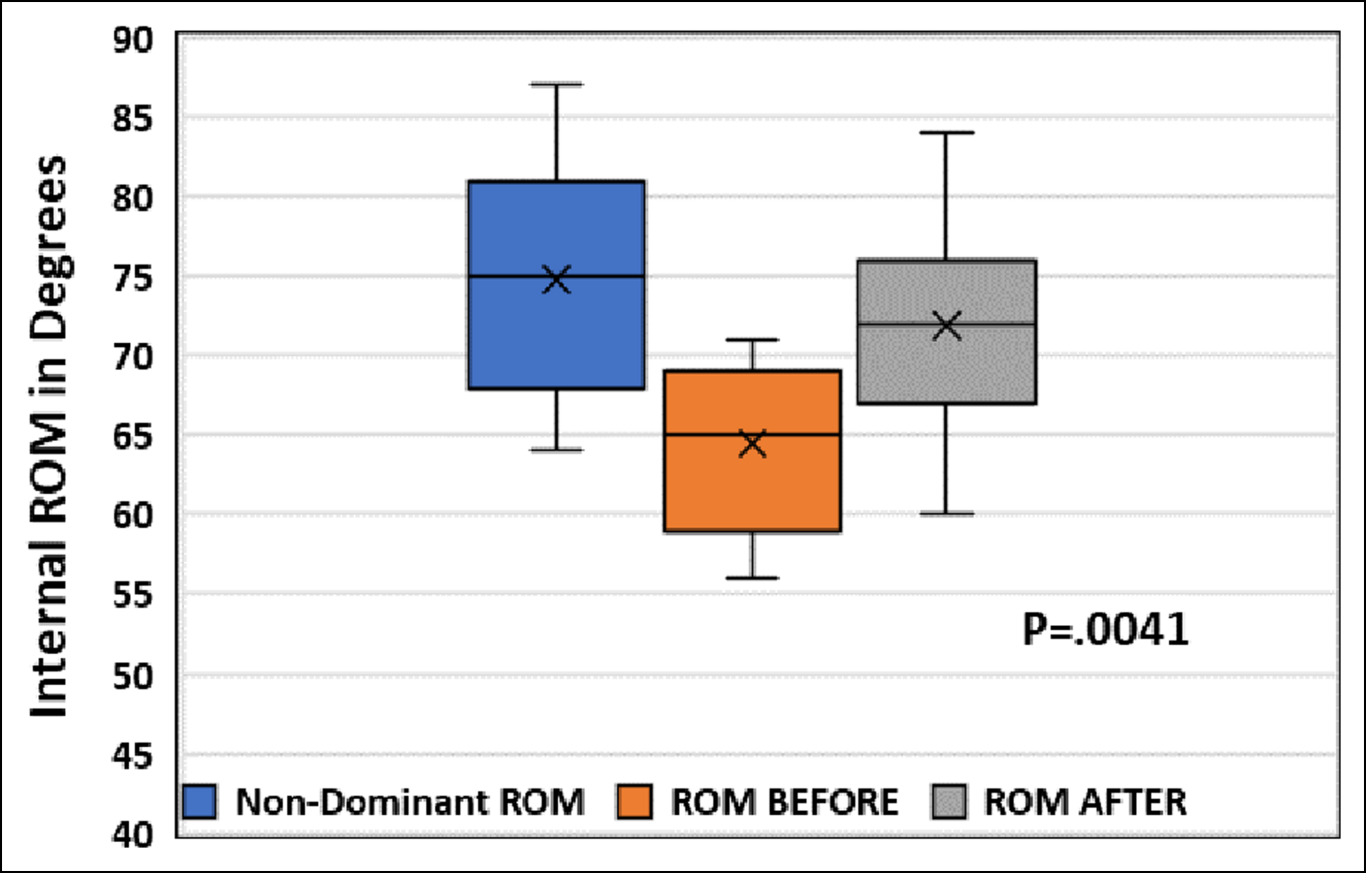
Box and whisker plot showing shoulder internal rotation of 15 minor league baseball players on the nondominant arm and dominant arm before and after SI joint stretch. SI = sacroiliac

**Figure 4:**
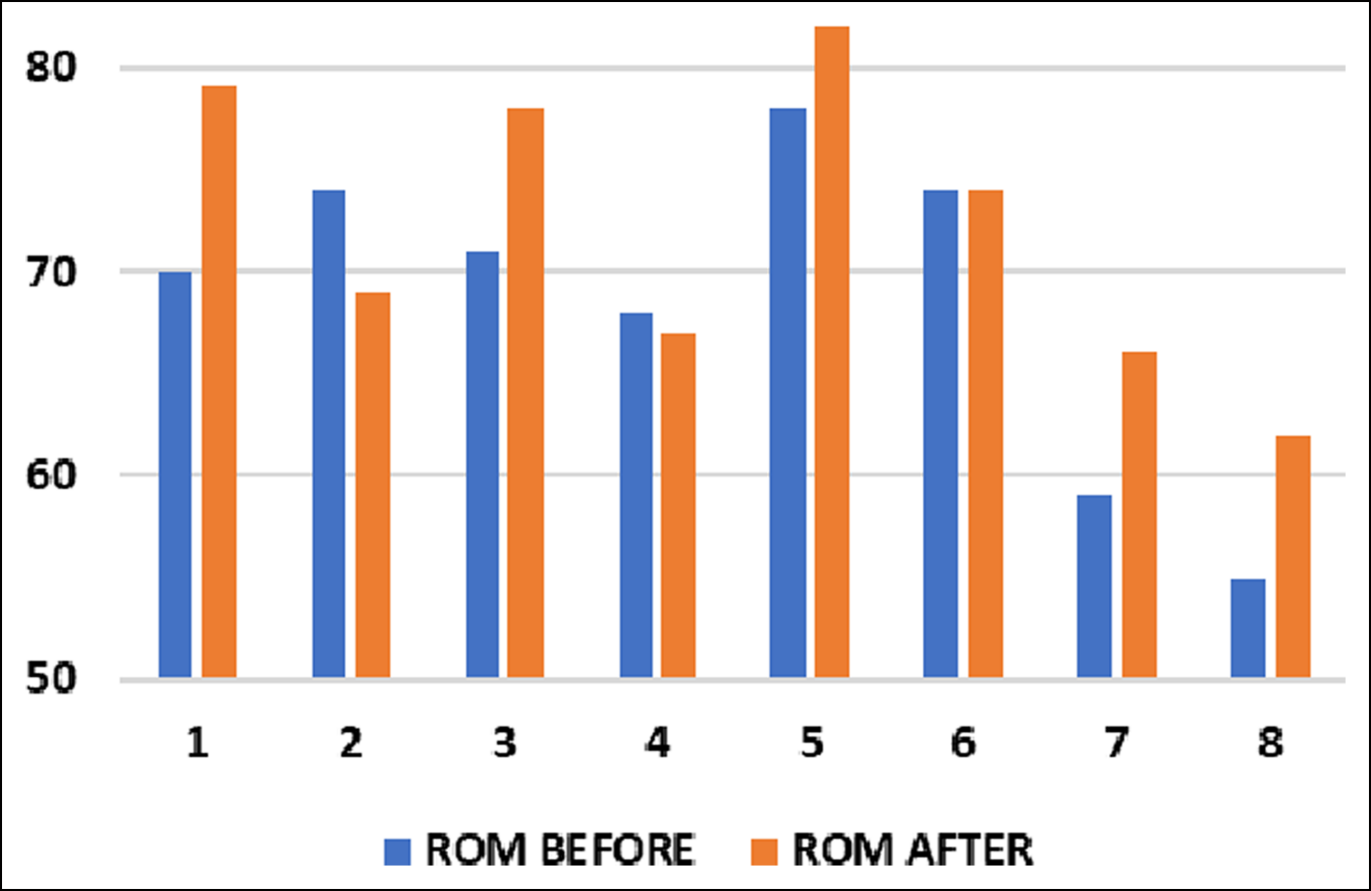
Graph showing shoulder internal rotation of the dominant arm before and after sleeper stretch.

**Figure 5:**
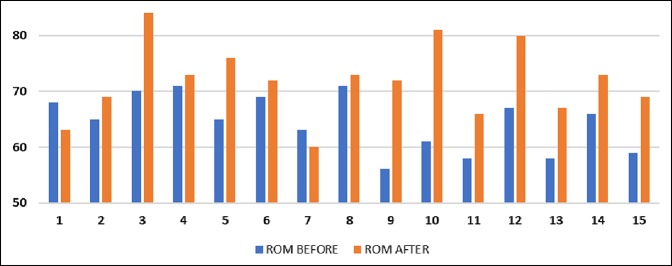
Box and whisker plot showing shoulder internal rotation of the dominant arm before and after SI joint stretch. SI = sacroiliac

After stretching, the sleeper stretch group's mean end-range IR was 72.1° (SD = 7.2°), a 3.5° improvement (*P* = 0.1058), whereas the contralateral SI joint stretch group's mean end-range IR was 71.9° (SD = 6.6°), a 7.4° improvement (*P* = 0.0041) (Figures 2–5). No significant difference was found between the contralateral SI joint stretch and sleeper stretch groups over time (*P* = 0.1978). No player was injured because of this study.

Intraclass correlation coefficients ranged from 0.47 to 0.87 for the right arm and 0.62 to 0.84 for the left arm, indicating moderate to good intrarater reliability^22^ of our procedure, with a standard error of measurement of 5.8° for the right arm and 5.3° for the left arm. Interrater reliability for measurements was 0.20 for the right arm and 0.40 for the left arm. Percent agreement was high (>85%), and percent chance agreement (81%). Mullaney et al^23^ report that using a digital leveler to measure the ROM of the shoulder is reliable, with a rotation measurement error of ±3° and an ability to detect differences in ROM of 6° with the same examiner and 15° between different examiners.

## Discussion

GIRD is a common physical finding in overhead athletes.^2,3,4,5^ Previous studies have demonstrated shoulder loss of motion after pitching that lasts more than 24 hours^.6,7^ Currently, the exact cause and underlying mechanism for this phenomenon are unclear. Pappas et al^24^ were the first to suggest that posterior shoulder stiffness results from repetitive microtrauma, leading to the development of fibrotic scar tissue of the posterior capsule. Reinold et al^6^ reported an immediate loss of rotation after pitching and speculated that this loss may be a normal physiologic response to muscle damage after high levels of eccentric contractions. Other authors have theorized that the loss of motion is the result of the cumulative effects of eccentric microtrauma to the posterior shoulder, leading to inflammation, scar formation, and subsequent tightness in the posterior glenohumeral capsule and posterior shoulder muscles, as well as osseous adaptations.^23-25^ If posterior capsular tightness were the singular cause of GIRD, one would expect alteration in posterior glenohumeral translation in pitchers. However, in an investigation of glenohumeral translation and shoulder mechanics in the setting of decreased IR, Borsa et al^4^ did not demonstrate any such translation. This study suggests that there may be other reasons that explain this loss of motion in overhead athletes.

In this study of asymptomatic minor league baseball players, we found that over half had measurable loss of shoulder motion compared with their contralateral shoulder. Many previous studies have offered treatment algorithms to improve shoulder ROM. Sleeper stretch and cross stretching most commonly have been shown to improve IR. Many experienced therapists and athletic trainers have developed their own treatment regimen of stretching. With this study, we attempted to validate scientifically what we had observed anecdotally about the relationship between SI joint tightness and posterior shoulder capsule tightness. Our results demonstrated that internal end-range rotation improved with the “benchmark” sleeper's stretch; however, rotation improved twice as much stretching the contralateral SI joint.

The bar graphs in Figures 3 and 4 help visualize the IR in each player before and after doing the sleeper and SI joint stretches, respectively. It should be noted that not every player improved with either stretches. We postulate that subjects who did not improve with the sleeper's stretch may have had a corresponding tightness in the pelvis and those who did not improve with the SI stretch may have had a physical contracture of the shoulder. Although capsular contracture is undoubtedly a component of GIRD in many patients, we hypothesize that there is a subset of players with GIRD who have pelvic tilting and will improve their shoulder range of motion with SI joint stretching.

Although tightness of the pelvis causing tightness of the shoulder is a new concept and much more research needs to be done to understand the reason behind this phenomenon, several studies show the correlation between loss of motion in the hip with loss of motion in the shoulder in pitchers from youth to professional baseball.^2,3,4,5,6,7,8,9,10,11,12,13,14,15,16,17,18,19,20,21,22,23,24,25,26,27,28,29,30,31,32^

Commonly, baseball players have been noted to possess decreased trunk rotation, shoulder IR of the throwing arm, external rotation of the dominant hip, and IR of the nondominant hip.^27,33^ Zeppieri et al^32^ demonstrated that the nondominant (lead) hip ROM and strength decreased over the course of a season. Picha et al^31^ found differences in ROM of both the hip and shoulder in youth baseball pitchers.

Sauers et al^29^ postulated that limitations in the hip motion of baseball players may lead to altered motion at the glenohumeral joint to maintain throwing velocity, thereby predisposing the upper extremity to injury. Oliver et al^30^ evaluated lower extremity ROM and upper extremity kinetics during baseball pitching in youth baseball pitchers. They found a “complex relationship” between the two.

At present, a paucity of literature exists to support the complex relationship between the lower back and GIRD. However, two studies investigated improvement in shoulder range of motion with rotating the trunk along with stretching the shoulder.^7,34^

Escamilla et al^7^ demonstrated that the immediate loss of motion after a 40-pitch bullpen session promptly returned to the prepitching range after a short-duration stretching/calisthenics “two-outs drill” (ie, various shoulder and elbow stretches and quick trunk rotational exercises). The personal investigator is speculating that the trunk rotational exercises restored the pitchers' pelvic tilt and, thus, contributed to the improved shoulder range of motion.

Gamma et al^34^ compared improvement in shoulder motion in two groups of pitchers—a traditional dynamic warm‐up group and a Total Motion Release (TMR) group. Tradition warm-up stretches included lunges, power skips, sprints, and sleeper stretch. TMR is a trademark program that evaluates and treats limitations in motion of the arms, legs, and trunk. Shoulder internal range of motion was significantly improved after TMR compared with the traditional warm-up (*P* = 0.025). Again, trunk rotational exercises are an integral part of the TMR program and also may have contributed to this significant improvement.

The pitching motion is a kinetic chain. In the past, most of the literature focused on the upper extremity in pitchers. MacWilliams et al^35^ pioneered the importance of the lower extremity in contributing in the throwing motion and suggested that strengthening of the lower extremities could enhance performance and avoid injury. The force generated by the large muscles of the lower extremity and trunk during the wind-up and stride phases are transferred to the ball through the shoulder and elbow during the cocking and acceleration phases.^36^ Breaks in this kinetic chain are known to increase the risk of injury.

Perhaps the great forces generated in the wind-up and stance phases throw the pelvis out of alignment, producing GIRD. If this phenomenon proves to be true, orthopaedic surgeons must not simply focus on the shoulder of overhead athletes with GIRD, getting mixed results, but also evaluate the lower back, looking for the true source of an injury, thus, obtaining quick, long-lasting relief and preventing future injuries. As the saying goes, “The trunk must be stable to support the limbs.”

## Limitations

There are several limitations to this study. First, the sample size is small, and the sizes between randomized groups are uneven. In addition, the cohort included both pitchers and position players. Future studies should attempt to study a more homogenous group. We also had measurements only for one point in time; thus, it is impossible to conclude whether the range-of-motion gains were maintained over time. We do not have any data on our study subjects regarding injuries, pain, or games missed due to pain/injury. We used a novel approach to our range of motion measurements that has not been validated in the literature. On the basis of this small pilot study, we recommend future research to confirm our findings. Because our study focused on only minor league baseball players, our findings may not be generalizable to other athletes or patient populations.

## Conclusion

On the basis of our data, there may be a subset of baseball players with a glenohumeral internal rotation deficit that is in part caused by SI joint tightness and may benefit or resolve from stretching the pelvis. Further anatomic and functional research studies are necessary to understand this pathophysiology.
